# Design and Fabrication of an Artificial Compound Eye for Multi-Spectral Imaging

**DOI:** 10.3390/mi10030208

**Published:** 2019-03-25

**Authors:** Axiu Cao, Hui Pang, Man Zhang, Lifang Shi, Qiling Deng, Song Hu

**Affiliations:** 1 Institute of Optics and Electronics, Chinese Academy of Sciences, Chengdu 610209, China; longazure@163.com (A.C.); ph@ioe.ac.cn (H.P.); zhangman881003@126.com (M.Z.); dengqiling@ioe.ac.cn (Q.D.); 2 University of Chinese Academy of Sciences, Beijing 100049, China

**Keywords:** artificial compound eye, multi-spectral imaging, lithography, spectral splitting

## Abstract

The artificial compound eye (ACE) structure is a new type of miniaturized, lightweight and intelligent imaging system. This paper has proposed to design a multi-spectral ACE structure to enable the structure to achieve multi-spectral information on the basis of imaging. The sub-eyes in the compound eye structure have been designed as diffractive beam splitting lenses with the same focal length of 20 mm, but with the different designed center wavelengths of 650 nm, 532 nm, and 445 nm, respectively. The proximity exposure lithography and reactive ion etching process were used to prepare the designed multi-spectral ACE structure, and the spectral splitting and multi-spectral imaging experiments were carried out to verify the multi-spectral imaging function of the structure without axial movement. Furthermore, the structure can be designed according to actual requirements, which can be applied to covert reconnaissance, camouflage identification, gas leakage or other fields.

## 1. Introduction

Compound eyes of arthropods such as ants, flies and bugs have attracted extensive research interests due to their unique features such as wide fields of view (FOV), high sensitivities to motions and infinite depths of field [1,2,3,4,5,6,7,8]. These compound eyes are integrated, lightweight and smart optical imaging systems composed of multiple individual lenses arranged on a hemispherical surface. Optical devices inspired by natural compound eyes exhibit great potentials in various applications such as surveillance cameras on micro aerial vehicles, high-speed motion detections, endoscopic medical tools, and image guided surgeries [9,10,11].

For years, several attempts were made to develop artificial compound eyes (ACEs) to realize imaging [12,13,14,15], which typically analyzes the spatial characteristics of the target by studying the shape information of the target. In this paper, the spectroscopy technology has been proposed to apply into the ACE structure combined with imaging. Based on the original two-dimensional spatial imaging, one-dimensional spectral information has been added in the structure. Compared with the traditional imaging technology, it can improve the accuracy and sensitivity of target detection, and expand the detection capability of traditional imaging systems for target detection, camouflage identification, complex background suppression, life state observation, environmental monitoring and other fields.

Multi-spectral imaging technology needs to divide the incident full-band or wide-band spectrum into several narrow-band spectrum so as to obtain images of different spectral bands by the corresponding detectors. Thereinto, spectral splitting technology is very important. Spectral splitting technology mainly includes interference spectroscopy [16,17] and traditional filter spectroscopy [18,19,20]. 

Interferometric imaging spectrometry detects the interferogram of target by combining two-beam interferometry with imaging technology and obtains spectral information by Fourier transform calculation. The structure of the system is complicated due to the addition of interferometric light paths. 

The traditional spectral imaging technology is that the narrow and rectangular slits are imaged on the two-dimensional detector by the prism or grating of the dispersive element. The dispersion direction of the prism or grating is perpendicular to the long axis of the slit. The detector records the spatial image in one-dimensional direction, and the spectral data in the other one-dimensional direction is the spatial spectral image. Then, the spectral data of the whole target is obtained by the push-sweep method. It is inconvenient to operate. The other traditional narrow-band tuned filter imaging spectroscopy technology uses the scheme of camera and filter. Its principle is simple and there are many kinds, such as circular gradient filter, liquid crystal tunable filter and filter wheel. This spectral imaging technology obtains monochrome image of scene object through one snapshot, but it needs to change or modulate the filter to obtain the whole image data. In general, current multispectral cameras are larger than normal cameras because they must be equipped with a mechanism to the original imaging system for changing the spectral transmittance, such as a prism, a grating or a filter wheel or a liquid crystal tunable filter.

ACE is suitable to be used as the lens of a multi-spectral imaging system because optical information of different bands can be captured simultaneously by different units. A typical multi-spectral imaging system based on ACE put a multi-channel color filter in front of the microlens array to realize spectral separation [21]. However, it will increase the complexity of the system and also bring the alignment problem between the ACE and the filter. The position mismatch of ACE and filters will make the imaging quality decline. Therefore, it is desirable to integrate the ACE with multi-channel filter in order to improving the system performance. Then, a compact structure of integrating a microlens array with a multi-channel filter based on a Micro-Electro-Mechanical-System (MEMS) technique has been fabricated [22]. However, the fabrication of multi-channel filter is complex because of the repeated fabrication process of multi-channel filter based on pigment-dispersed method.

The diffractive beam splitting lenses can realize the function of imaging and spectroscopy at the same time. Diffraction optical spectrum imaging is a staring imaging technology based on the diffractive beam splitting lenses, which can simultaneously image the scene in the field of view and read it out by using a focal plane array detector at one time. Therefore, the structure is more compact and the optical path is much simpler because there is no optical-mechanical scanning component. At the same time, the whole input aperture can collect light, which is contrary to the narrow input slit of traditional spectral imaging. The luminous flux of the system is much higher than that of conventional dispersive spectral imaging technology. Meanwhile, diffraction optical spectrum imaging technology is very flexible. It can collect all or part of the band information by program control in the free band of the instrument. It can be used for spectrum detection from ultraviolet to far infrared band, which is limited only by detector array technology.

Generally, diffraction optical spectrum imaging has the advantages of compact structure, high throughput and staring imaging [23,24]. However, in the image space, the axial distance adjusting device should be used to adjust the detector to move along the optical axis; then, the corresponding two-dimensional spectral image sequence can be collected to achieve multi-spectral imaging. 

In this paper, on the basis of making full use of the spectral imaging advantages of diffractive beam splitting lenses, different sub-eye imaging channels of the compound eye are designed to capture spectral information of different bands at the same distance avoiding the use of axial distance adjusting device, which can effectively reduce the dependence of the acquisition of different spectral information on the distance between the lens and the detector so that the ACEs can realize multi-spectral imaging function on the basis of imaging, which can be used in concealment reconnaissance, camouflage recognition, toxic gas leakage and other fields.

## 2. Principle and Structural Design

**Figure 1:**
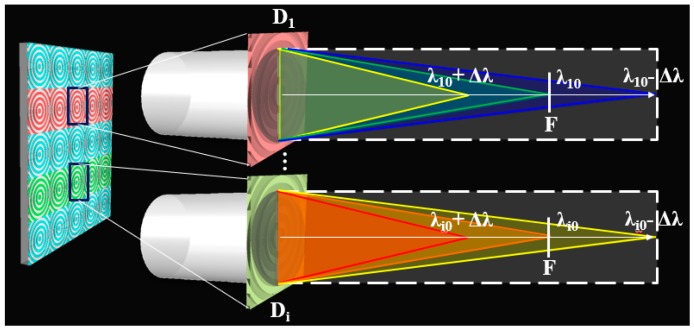
Multi-spectral imaging principle of artificial compound eye (ACE).

The ACE is composed of several sub-eye imaging channels, as shown in Figure 1. Different sub-eye imaging channels are designed as diffractive beam splitting lenses (D_i_) with different designed parameters. The lenses have the same focal length (*F*) and different central wavelength ($\lambda_{i{}_{0}}$). In the imaging process, spectral information of different wavelengths can be captured by different sub-eyes at the same image plane.

**Figure 2:**
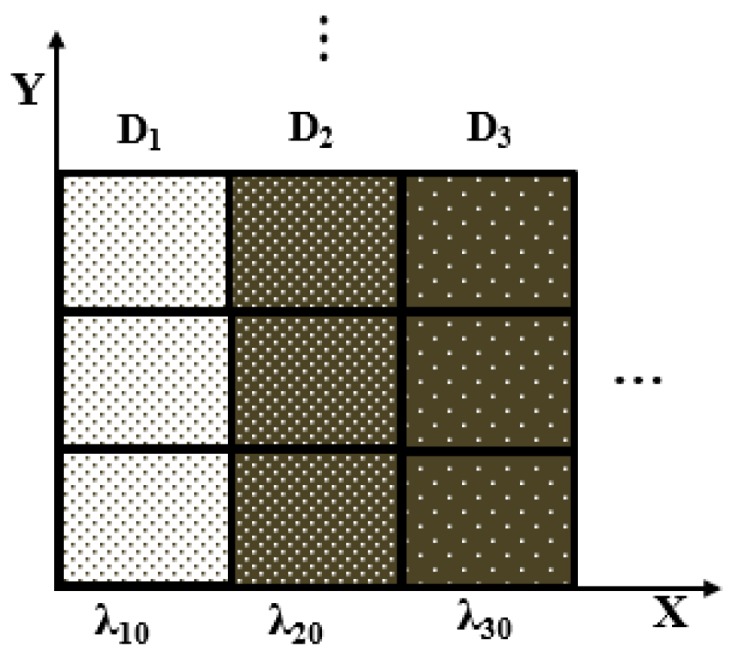
The arrangement of sub-eye lenses in multi-spectral ACE.

The arrangement of the sub-eye lenses in the multi-spectral ACE is shown in Figure 2. Along the Y direction, the sub-eye lenses are the same. In the X direction, the sub-eye lenses are designed for different wavelengths. The designed wavelengths ($\lambda_{i{}_{0}}$, *i* = 1,2,3) are 650 nm (*R*), 532 nm (*G*) and 445 nm (*B*), respectively. The aperture (*D_i_*, *i* = 1,2,3) are the same as 2 mm, and the focal length (*F*) is also the same as 20 mm. Three sub-eye lenses with different central wavelengths are arranged periodically in the X direction with a period of 6 mm to form a complete ACE structure.

Different sub-eyes are composed of different annular structures. The maximum number of annular bands of the corresponding sub-eye lenses is determined by the aperture (*D_i_*) of the sub-eye lens, the design wavelength ($\lambda_{i{}_{0}}$) and the focal length (*F*), which can be calculated by Formula (1). Then, according to Formula (2), the radius ($r_{i{}_{n}}$) corresponding to different number of annular bands ($n_{i}$) can be calculated to obtain the complete distribution of annular bands structure for different sub-eye lenses, and some radius values are shown in Table 1:

(1) $$N_{i}=\frac{{\left(D_{i}/2\right)}^{2}}{F\lambda_{i0}},i=1,2,3;$$

(2) $$r_{i}{}_{n}=\sqrt{n_{i}F\lambda_{i0}},n_{i}=1,2\cdots N_{i};$$

## 3. Experiments and Discussion

### 3.1. Fabrication

The preparation technology of the proposed multi-spectral ACE was studied. The preparation process of diffractive beam splitting lens with a central wavelength of 532 nm is described in detail below. When the central wavelength is 532 nm, the etching depth (*h*) can be obtained by Formula (3). The refractive index (*n*) of silica material is 1.461 at 532 nm, so the etching depth is calculated as 577 nm:

(3) $$h=\frac{\lambda }{2(n-1)},n=1.461@532\;nm;$$

**Figure 3:**
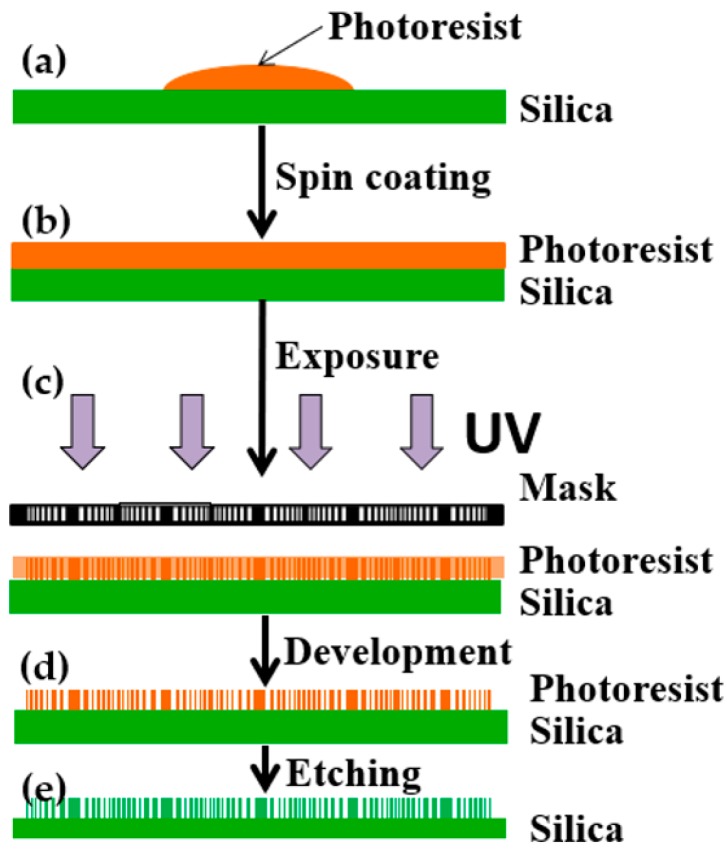
Preparation technology of multi-spectral ACE: (**a**) substrate preparation; (**b**) photoresist was spin-coated on the silica; (**c**) proximity exposure with designed mask; (**d**) photoresist-based structure was achieved after development; (**e**) the pattern was transferred to silica by etching.

**Figure 4:**
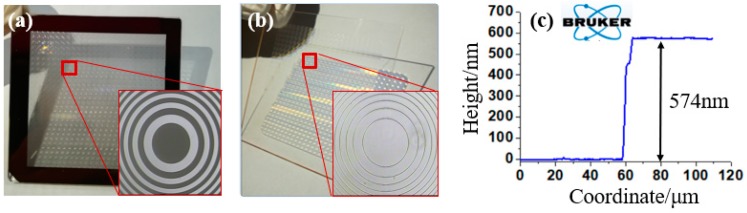
Key fabrication results, including: (**a**) mask; (**b**) prototype based on silica; (**c**) measurement of the fabricated structure by a step proflometer.

The fabrication process flow mainly includes photoresist spin coating, exposure, development and etching, as shown in Figure 3. Firstly, the silica material (JGS1) was selected as the substrate material, and the photoresist AZ1500 (20cp) (AZ 1500 Photoresist (20cp), Merck Electronic Materials, Shanghai, China) was spun on the surface of the substrate at a speed of 6000 rpm for 30 s as the initial structure (Figure 3a,b). Key parameters such as prebake temperature and prebake period were 90 °C and 10 min, respectively. Secondly, the annular bands structure was encoded as the mask data according to the design parameters, and the mask was prepared using the high-precision laser direct writing technology, as shown in Figure 4a. The UV lithography machine (URE-2000S/A) with a central wavelength of 365 nm was used. The exposure mode was proximity exposure with total exposure time of 10 s at an exposure power density of 2.5 mW/cm^2^ (Figure 3c). Third, the development and post-exposure bake were carried out. The photoresist was developed in a solution of AZ300MIF (AZ300MIF Developer, Merck Electronic Materials, Shanghai, China) for 25 s, followed by 2 h post-exposure bakes at 120 °C, and the photoresist multi-spectral compound eye structure was obtained as shown in Figure 3d. Finally, etching of the silica was conducted, which transferred the patterns of the multi-spectral compound eye structure to the silica substrate (see Figure 4b). A reactive ion etching machine was used to transfer the structure into the substrate. The etching gases were SF_6_ and CHF_3_ in the proportion of 1:20 and the etching time was 15 min (see Figure 3e).

A step profilometer (Stylus Profiler System, Dektak XT, Bruker, Karlsruhe, Germany) was used for the measurement of the fabricated structure, and the cross-sectional profile was drawn by the scientific drawing and data analysis software of OriginLab (OriginPro 8.0, Northampton, MA, USA) with etching depth of 574 nm (see Figure 4c), which was consistent with the design result.

The structure of different diffractive beam splitting lenses corresponding to different wavelength was realized by multiple overlay exposure and etching technology. The lenses with different depths were obtained by changing etching times during the etching process.

The diffraction efficiency of the structure is related to the step number of the actual structure. The higher the step number is, the higher the diffraction efficiency is. When the number of steps is 2, the diffraction efficiency is about 40%; when the number of steps is 4, the diffraction efficiency is more than 80%; when the number of steps is more than 8, the diffraction efficiency can be more than 90%; if the number of steps is 32, the diffraction efficiency can be as high as 99%. Of course, with the increase of the number of processing steps, the difficulty of preparation process will also increase. In order to verify the principle of the proposed structure from the optical effect, the structure of the fabricated structure is only two steps, and the diffraction efficiency is slightly low at 40%. If the practical application of the structure requires higher diffraction efficiency, the structure with higher steps can be prepared.

### 3.2. Imaging Verification

**Figure 5:**
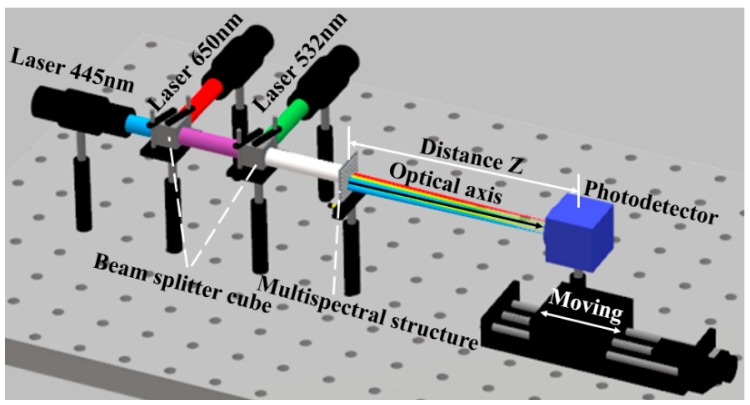
The light path diagram of red, green and blue laser beam combined coaxially illuminate the multi-spectral compound eye structure.

The imaging light path was constructed to verify the spectral characteristics of the prepared multi-spectral ACE structure. The structure was irradiated by red, green and blue lasers of 650 nm, 532 nm and 445 nm, which were consistent with the design center wavelength, as shown in Figure 5. During the experiment, the expanding and collimation of the laser beam were firstly realized, so that the laser beam with a diameter of more than 6 mm can simultaneously illuminate the area with different designed center wavelengths on the multi-spectral compound eye structure. Secondly, the three-color beams were combined to make the three-channel beam coaxially illuminate the multi-spectral compound eye structure. Finally, by adjusting the distance Z between the photodetector and the structure, the spot distributions of different wavelengths at different distances were observed.

**Figure 6:**
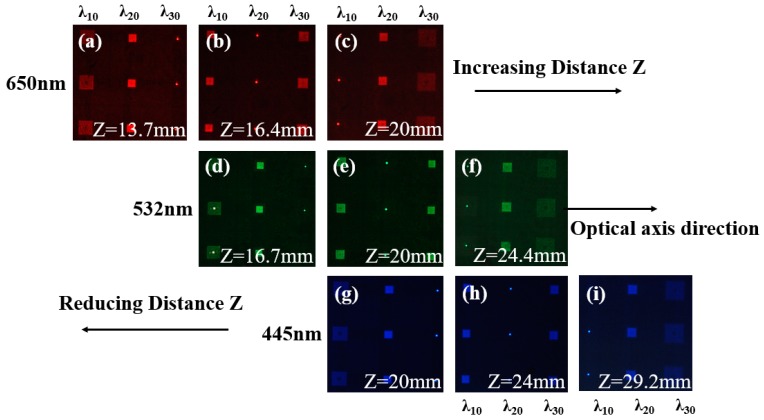
The spot distributions of red, green and blue laser after multi-spectral compound eye structure: (**a**–**c**) are the spots at the distance of 13.7 mm, 16.4 mm and 20 mm when the red laser beam with the wavelength of 650 nm irradiates through the structure, respectively; (**d**–**f**) are the spots at the distance of 16.7 mm, 20 mm and 24.4 mm when the green laser beam with the wavelength of 532 nm irradiates through the structure, respectively; (**g**–**i**) are the spots at the distance of 20 mm, 24 mm and 29.2 mm when the blue laser beam with the wavelength of 445 nm irradiates through the structure, respectively.

During the experiment, three laser beams were independently used to illuminate the multi-spectral compound eye structure. At the design focal length of 20 mm, laser beams of different wavelengths were converged by different sub-eye lenses, as shown in Figure 6. The sub-eye lens structure marked with λ_10_ corresponds to the designed wavelength of 650 nm, so when the red laser beam irradiated the structure, the detector detected the focus at 20 mm converged by the corresponding sub-eye structure, as shown in Figure 6c.

Accordingly, when the green laser beam irradiated the structure with λ_2__0_ corresponding to the designed wavelength of 532 nm, the detector detected the focus at 20 mm converged by the corresponding sub-eye structure, as shown in Figure 6e. When the blue laser beam irradiated the structure with λ_3__0_ corresponding to the designed wavelength of 445 nm, the detector detected the focus at 20 mm converged by the corresponding sub-eye structure, as shown in Figure 6g.

**Figure 7:**
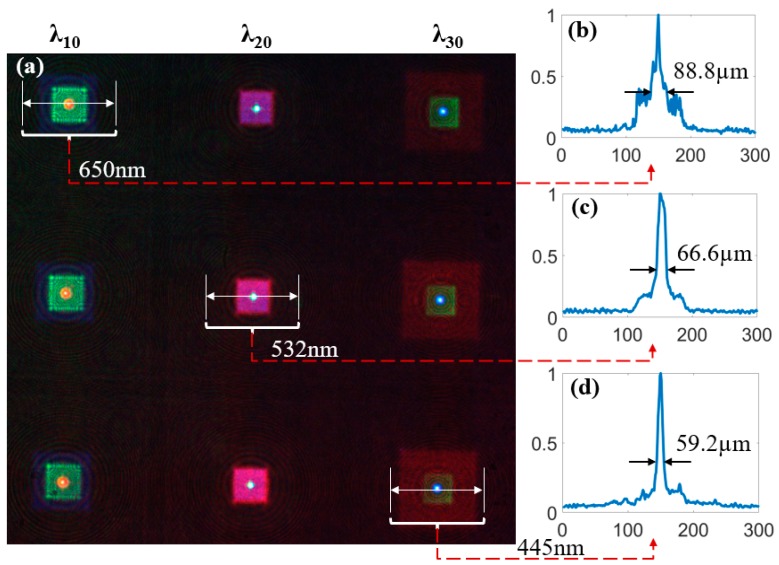
Distributions of the focal spots: (**a**) distributions of focal spots when red, green and blue lasers simultaneously irradiate the multi-spectral compound eye structure; (**b**) the intensity distribution of the focal spot at a wavelength of 650 nm; (**c**) the intensity distribution of the focal spot at a wavelength of 532 nm; (**d**) the intensity distribution of the focal spot at a wavelength of 445 nm.

When tricolor laser beams of red, green and blue illuminate the multispectral structure at the same time, it can be seen that different laser beams of different wavelengths can be converged by the corresponding sub-eye structures of the same designed wavelengths, while the other laser beams disperse, as shown in Figure 7a. It is verified that the structure can realize the spectral splitting function for different wavelengths by different sub-eye imaging channels without axial movement. The intensity distributions of the focal spot sizes were analyzed and shown in Figure 7b–d. The ranges of the energy of the focal spots reduced from the highest to 1/e were calculated as the sizes of the focal spots. The focal spot sizes of the wavelengths of 650 nm, 532 nm and 445 nm were quantified as 88.8 μm, 66.6 μm and 59.2 μm, respectively. 

Under the same focal length and aperture, the longer the wavelength is, the larger the focal spot size is. The experimental results are in agreement with the theory. Therefore, with the increase of focal spot size, the imaging quality will become worse. In addition, because the scattered spots of other wavelengths are superimposed on the focal spots, the sizes of the captured focal spots which affects the imaging quality are larger than that of the theoretical Airy spots. From the energy distribution curve, it can be seen that, for the focal spot of red light, the intensity of the diffuse spots produced by other wavelengths is larger than the other colors. This is also the reason for the poor effect of red image in the later imaging experiment.

At the same time, if the detector is moved axially, the spectral splitting function of each sub-eye lens can still be observed. For example, a sub-eye lens with a central wavelength of λ_10_ can converge red light of 650 nm at the distance of 20 mm, as shown in Figure 6c. With the increase of distance Z, the sub-eye lens converges green light at the distance of 24.4 mm, as shown in Figure 6f. As the distance Z increases more, the blue light converges at the distance of 29.2 mm, as shown in Figure 6i. This is determined by the spectral splitting characteristics of the sub-eye lens. Its focal length is inversely proportional to the wavelength. With the increase of distance, the wavelength of the converged light decreases. The wavelength decreases from 650 nm to 532 nm, and then to 445 nm in the experiment, which is consistent with the spectral splitting theory.

Similarly, the sub-eye lens with a central wavelength of λ_2__0_ can converge green light of 532 nm at the distance of 20 mm, as shown in Figure 6e, With the increase of distance Z, the sub-eye lens converges shorter wavelength of blue light at the distance of 24 mm, as shown in Figure 6h. With the decrease of distance Z, the sub-eye lens converges longer wavelength of red light at the distance of 16.4 mm, as shown in Figure 6b. A sub-eye lens with a central wavelength of λ_3__0_ can converge blue light of 445 nm at a distance of 20 mm, as shown in Figure 6g. With the decrease of distance Z, sub-eye lens converges longer wavelength green light at the distance of 16.7 mm, as shown in Figure 6d. With the more decrease of distance Z, sub-eye lens converges the longest wavelength red light at the distance of 13.7 mm, as shown in Figure 6a.

**Figure 8:**
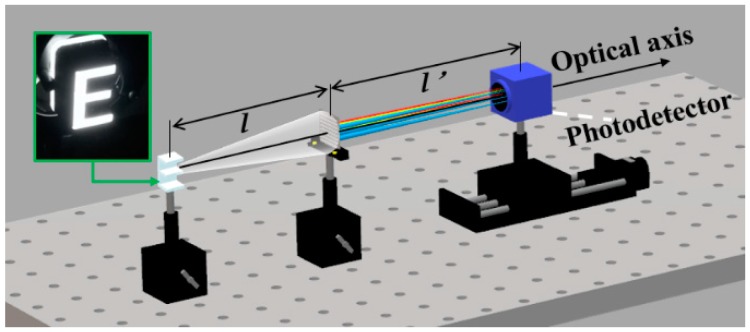
Multi-spectral imaging experimental verification.

**Figure 9:**
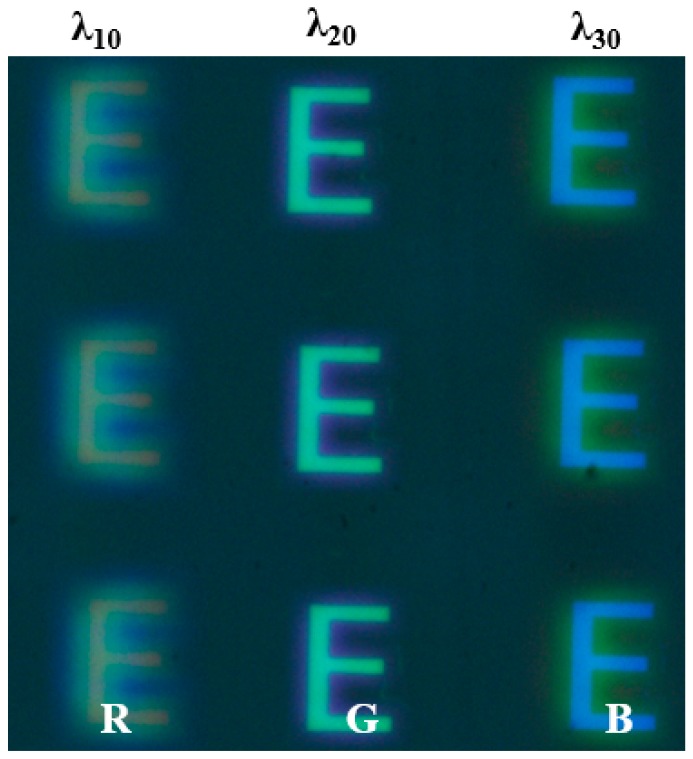
Red, Green and Blue multi-spectral imaging effects.

Furthermore, in order to verify the imaging characteristics of the red, green and blue multi-spectral compound eye structure, the prepared structure was used to image the luminous object “E”. The luminous object was white light in a wide band, and the image was captured by the photodetector. The overall experimental light path was shown in Figure 8. The object distance (*l*) between “E” and multi-spectral compound eye structure was set as 1850 mm. The distance between the detector and the multi-spectral compound eye structure was defined as the imaging distance (*l’*). According to the Gauss imaging formula, the imaging distance was calculated as 20.22 mm with the focal length of 20 mm for various wavelengths. The detector was moved along the axis to record the image, which was located on the corresponding imaging plane of the structure. The imaging effect of the prepared structure was effectively verified as shown in Figure 9. The detector obtained different wavelengths of red, green and blue spectral information at the same image plane, which verified the multi-spectral imaging performance of compound eye structure. It is worth mentioning that the spectral image received by the detector is the overlap image of the focus image formed by one wavelength on its focal plane and defocus images of other wavelengths at this position. Therefore, the light from other spectral channels will cause interference and blur to the image. A series of algorithms are needed to process the image in the following steps to obtain the target spectral image data cube. In order to eliminate spectral blurring and restore image quality, image space linearization based on a translation invariant model is needed, that is, the relative wavelength of spectral image should have an equal interval and equal frame. Then, the three-dimensional deconvolution and other reconstruction image processing techniques in three-dimensional optical slice microscopy technology need to be used to obtain clear spectral image information.

## 4. Conclusions

In this paper, a new multi-spectral ACE structure has been designed and fabricated by combining the diffractive beam splitting lenses with the ACE structure. Experiments of spectral splitting and imaging were carried out, and the red, green and blue spectral splitting and imaging were realized, which verified the multi-spectral imaging function of the structure. Because the ACE structure is a multi-aperture imaging structure composed of multiple sub-eye lenses, spectral imaging structures with different central wavelengths can be designed according to the needs. 

## Figures and Tables

**Table 1:** Radius values of multi-spectral structure in different annular bands.

n	*r*_1n_/µm	*r*_2n_/µm	*r*_3n_/µm
1	114.0175	103.1504	94.33981
2	161.2452	145.8767	133.4166
3	197.4842	178.6617	163.4013
4	228.0351	206.3008	188.6796
5	254.9510	230.6513	210.9502
6	279.2848	252.6658	231.0844
7	301.6621	272.9102	249.5997
8	322.4903	291.7533	266.8333
9	342.0526	309.4511	283.0194
10	360.5551	326.1901	298.3287
11	378.1534	342.1111	312.8898
12	394.9684	357.3234	326.8027
13	411.0961	371.9140	340.1470
14	426.6146	385.9534	352.9873
15	441.588	399.4997	365.3765
16	456.0702	412.6015	377.3592
17	470.1064	425.2999	388.9730
18	483.7355	437.6300	400.2499
19	496.9909	449.6221	411.2177
20	509.902	461.3025	421.9005
…	…	…	…

## References

[1] Jeong K.H., Kim J., Lee L.P. (2006). Biologically inspired artificial compound eye. Science.

[2] Luo J., Guo Y., Wang X., Fan F. (2017). Design and fabrication of a multi-focusing artificial compound eyes with negative meniscus substrate. J. Micromech. Microeng..

[3] Cao A., Shi L., Shi R., Deng Q., Du C. (2012). Image process technique used in a large FOV compound eye imaging system. Proc. SPIE.

[4] Song Y.M., Xie Y., Malyarchuk V., Xiao J., Jung I., Choi K.J., Lu C., Kim R.-H., Li R., Crozier K.B. (2013). Digital cameras with designs inspired by the arthropod eye. Nature.

[5] Jian H., He J., Jin X., Chen X., Wang K. (2017). Automatic geometric calibration and three-dimensional detecting with an artificial compound eye. Appl. Opt..

[6] Li L., Hao Y., Xu J., Liu F., Lu J. (2018). The Design and Positioning Method of a Flexible Zoom Artificial Compound Eye. Micromachines.

[7] Kuo W.K., Lin S.Y., Hsu S.W., Yu H.H. (2017). Fabrication and investigation of the bionic curved visual microlens array films. Opt. Mater..

[8] Moghimi M.J., Fernandes J., Kanhere A., Jiang H. (2015). Micro-Fresnel-Zone-Plate Array on Flexible Substrate for Large Field-of-View and Focus Scanning. Sci. Rep..

[9] Yoshimoto K., Yamada K., Watabe K., Kido M., Nagakura T., Takahashi H., Nishida T., Iijima H., Tsujii M., Takehara T. (2015). Gastrointestinal tract volume measurement method using a compound eye type endoscope. Proc. SPIE.

[10] Kagawa K., Horisaki R., Ogura Y., Tanida J. (2009). A compact shape-measurement module based on a thin compound-eye camera with multiwavelength diffractive pattern projection for intraoral diagnosis. Proc. SPIE.

[11] Kaadan A., Refai H., Lopresti P. (2016). Wide-area and omnidirectional optical detector arrays using modular optical elements. Appl. Opt..

[12] Lin J., Kan Y., Jing X., Lu M. (2018). Design and Fabrication of a Three-Dimensional Artificial Compound Eye Using Two-Photon Polymerization. Micromachines.

[13] Liu F., Diao X., Li L., Hao Y., Jiao Z. (2018). Fabrication and Characterization of Inhomogeneous Curved Artificial Compound Eye. Micromachines.

[14] Cao A., Wang J., Pang H., Zhang M., Shi L., Deng Q. (2018). Design and fabrication of a multifocal bionic compound eye for imaging. Bioinspir. Biomim..

[15] Cao A., Shi L., Deng Q., Pang H., Man Z., Du C. (2015). Structural design and image processing of a spherical artificial compound eye. Optik.

[16] Sánchez F.M., Gál C., Eisenhauer F., Krabbe A., Haug M., Iserlohe C., Herbst T.M. (2008). LIINUS/SERPIL: A design study for interferometric imaging spectroscopy at the LBT. Proc. SPIE.

[17] Edelstein J. (1996). Imaging interferometric spectroscopy for advanced missions. Spies International Symposium on Optical Science. Proc. SPIE.

[18] Zhang Y.H., Yang H.M., Kong C.H. (2014). Spectral imaging system on laser scanning confocal microscopy. Opt. Precis. Eng..

[19] Wu W.-D. (2009). Spectral characteristics of displacement for parallel beam splitting prisms. Laser Technol..

[20] Ura S., Sasaki T., Nishihara H. (2001). Combination of grating lenses for color splitting and imaging. Appl. Opt..

[21] Shogenji R., Kitamura Y., Yamada K., Miyatake S., Tanida J. (2004). Multispectral imaging using compact compound optics. Opt. Express.

[22] Jin J., Di S., Yao Y., Du R., Du R. (2013). Design and fabrication of filtering artificial-compound-eye and its application in multispectral imaging. Proc. SPIE.

[23] Hinnrichs M., Massie M.A. (1997). New approach to imaging spectroscopy using diffractive optics. Proc. SPIE.

[24] Hinnrichs M. (2011). Simultaneous multispectral framing infrared camera using an embedded diffractive optical lenslet array. Proc. SPIE.

